# Shear Wave Dispersion Slope Measured with Shear Wave Dispersion Imaging Is Associated with Variceal Hemorrhage in Cirrhotic Patients

**DOI:** 10.3390/diagnostics12122909

**Published:** 2022-11-23

**Authors:** Xiaohui Sun, Li Zhang, Ling Jiang, Ligang Cui, Xiaoguang Li

**Affiliations:** 1 Department of Ultrasound, Peking University Third Hospital, Beijing 100191, China; 2 Department of Gastroenterology, Peking University Third Hospital, Beijing 100191, China; 3 Department of Infectious Diseases, Peking University Third Hospital, Beijing 100191, China

**Keywords:** ultrasound, liver stiffness, shear wave dispersion slope, cirrhosis, variceal hemorrhage

## Abstract

Background and Objectives: Portal hypertension (PH), as the main consequence of cirrhosis, leads to the development of gastroesophageal varices (GEVs). Variceal hemorrhage (VH) caused by the rupture of GEVs is a life-threatening emergency. Thus, the prediction of VH risk is considerably important. Our pilot study aimed to identify the risk factors of variceal hemorrhage (VH) in cirrhosis. Materials and Methods: Cirrhotic patients were prospectively included and divided into two groups according to the presence or absence of VH. Conventional ultrasound and shear wave dispersion (SWD) imaging were conducted to detect the portal vein diameter, spleen diameter, ascites, liver stiffness (LS) and shear wave dispersion slope (SWDS). The laboratory tests were recorded, including platelets (PLT), alanine transaminase (ALT), aspartate aminotransferase (AST), total bilirubin (TBIL) and albumin (ALB). The risk factors of VH were screened using univariate analyses and identified using multivariate logistic regression. The ROC curves were used to assess diagnostic accuracy. Comparisons between AUCs were performed using the Delong method. Results: Sixty-five patients with 22 VHs were finally included. The SWDS, spleen diameter and ascites were identified as independent risk factors for VH. The SWDS showed good performance for diagnosing VH (AUC = 0.768, 95% CI: 0.647–0.864), and sensitively identified 95.5% (95% CI: 77.2%–99.9%) of patients with VH. Including the three risk factors in multivariate logistic regression, we obtained a formula for diagnosing VH: −20.749 + 0.804 × SWDS + 0.449 × spleen diameter + 1.803 × ascites (no ascites = 0, ascites = 1). Comparison of AUCs revealed that the formula (AUC = 0.900, 95% CI: 0.800–0.961) performed better than LS, SWDS, and spleen diameter in diagnosing VH (*p* < 0.001; *p* < 0.05; *p* < 0.05). Conclusions: SWDS is a sensitive parameter for assessing the risk of VH. Combining the SWDS, spleen diameter and ascites resulted in good diagnostic accuracy.

## 1. Introduction

Portal hypertension (PH), as the main consequence of cirrhosis, contributes to the development of collateral circulation, of which gastroesophageal varices (GEVs) are the most clinically relevant portosystemic collateral. PH is evaluated by the hepatic vein pressure gradient (HVPG). Clinically significant PH (CSPH) is defined when HVPG ≥ 10 mmHg, which is necessary for the development of GEVs and other complications, such as ascites and variceal hemorrhage (VH). GEVs occur in approximately 50% of cirrhotic patients [1]. VH caused by the rupture of GEVs is a life-threatening emergency with a six-week mortality of up to 20% [2]. 

As VH is a dangerous complication directly related to death, the prediction of VH risk is considerably important for improving the clinical prognosis. Although HVPG is the gold standard method for assessing PH, it is invasive and only available in top medical centers. Upper gastrointestinal endoscopy is recommended for evaluating the presence and size of GEVs. However, endoscopy is an invasive procedure, with the rare but serious complication of bleeding, and poor patient compliance. Therefore, non-invasive approaches for evaluating GEVs need to be explored. 

Shear wave dispersion (SWD) imaging is emerging as a new technique for measuring shear wave dispersion slope (SWDS). The SWDS is a viscosity-related parameter [3,4]. To our knowledge, no published study has examined the value of SWDS in diagnosing VH. First, this study aimed to investigate whether the SWDS is associated with the presence of VH. Second, we aimed to construct a regression-based formula and assess its diagnostic performance for VH.

## 2. Materials and Methods

### 2.1. Patients

The study was approved by the Medical Science Research Ethics Committee of our hospital, and written informed consent was obtained from all patients. We prospectively enrolled 65 patients with cirrhosis in this study. Cirrhosis was diagnosed based on either histologic findings or combined clinical, endoscopic, biochemical, and radiologic findings. The exclusion criteria were as follows: hepatocellular carcinoma; portal vein thrombosis or tumor thrombus; previous surgery for PH (including transjugular intrahepatic portosystemic shunt, splenectomy, or splenic embolization); upper gastrointestinal bleeding resulting from causes other than variceal rupture; and unreliable measurement of viscosity or elasticity with interquartile range (IQR)/Median > 0.3. The participants with cirrhosis were divided into non-VH (*n* = 43) and VH (*n* = 22) groups based on clinical and endoscopic findings. Endoscopy was performed within 24 h after presentation with upper gastrointestinal bleeding. The diagnosis of VH was based on the endoscopic signs of recent hemorrhage, such as active bleeding, a platelet plug, or overlying clot [5]. When no clinical symptoms of upper gastrointestinal bleeding occurred, such as hematemesis and melena, participants were classified as non-VH group.

### 2.2. Ultrasound Examination

**Figure 1:**
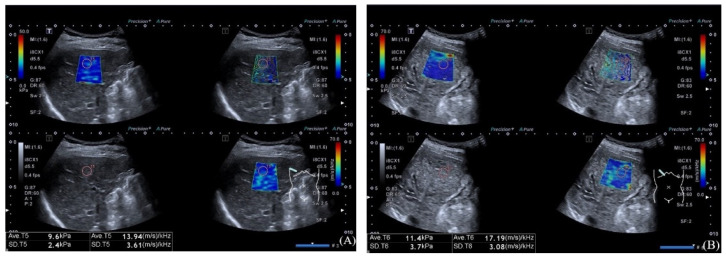
Four maps include an elasticity map (upper left), propagation map (upper right), B-mode map (lower left) and SWDS map (lower right): (**A**) images in a 50-year-old man who suffers hepatis B virus-related cirrhosis without variceal hemorrhage; and (**B**) images in a 52-year-old woman who suffers primary biliary cholangitis with variceal hemorrhage.

Ultrasound examination was conducted by one board-certificated radiologist using a US scanner (Aplio i900; Canon Medical System, Otawara, Japan) with a 1–8 MHz convex probe. Before the US examination, all participants were asked to fast for at least 4 h. The examination procedure was performed with participants in the supine position with the right arms extended above the head. First, we used B mode to detect the portal vein diameter, spleen diameter and ascites. The spleen diameter was defined as the bipolar diameter of the spleen crossing the spleen hilum. Excluding other causes of ascites such as heart failure, it was considered as positive regardless of the amount of ascites. Next, the probe was placed in the right intercostal space to obtain a proper ultrasound window, and the participants were requested to hold their breath. Then, two-dimensional shear wave elastography (2-D SWE) was conducted through “multi-mode”, and a 3 × 3-cm sized sample box was placed on the liver parenchyma at least 1 cm beneath the liver capsule to avoid reverberation artifacts. After the acquisition of shear wave propagation using acoustic radiation force, we froze the image and switched the twin view to a quad view including the B-mode image, propagation map, elasticity map and SWDS map (Figure 1). One-cm-sized circular regions of interest (ROI) were placed in the sample box within a 5 cm depth. We measured the LS value and SWDS value at least five times, and the median values were used for analysis. Reliable measurements were defined when the ratio of the IQR to the median value below 0.3.

### 2.3. Laboratory Test

All patients underwent laboratory test and ultrasound examination with an internal within less than one week. The laboratory tests included platelets (PLT), alanine transaminase (ALT), aspartate aminotransferase (AST), total bilirubin (TBIL), and albumin (ALB).

### 2.4. Statistical Analysis

We analyzed the normal or non-normal distribution of quantitative variables using the Shapiro–Wilk test. Quantitative variables were expressed as mean and standard deviation or median and IQR. The quantitative variables were compared using the independent t test or Mann–Whitney U test, when appropriate. Qualitative variables were expressed as number and percentage. Comparisons between categorical variables were performed by using χ^2^ test or Fisher’s exact test, when appropriate. Variables with *p* < 0.05 in univariate analysis were included in the multivariate analysis, and binary logistic regression analysis was used to determine the independent factors associated with VH. Factors with *p* < 0.05 were selected as components of the new formula for VH diagnosis.

The receiver operating characteristic (ROC) curves were constructed to assess the diagnostic accuracy of different variables for VH. The area under ROC curve (AUC), cut-off value, sensitivity, specificity, positive predictive value (PPV), and negative predictive value (NPV) were computed. The AUCs were compared using the Delong method. 

A significant level of two-side *p* < 0.05 was chosen. Statistical analysis was performed using SPSS (Version 23.0, IBM Corp, Armonk, NY, USA) and MedCalc (Version 20.0.3, MedCalc Software bvba, Ostend, Belgium).

## 3. Results

The study group finally enrolled 38 men and 27 women. Their mean age was 56.6 (±11.9) years. The etiologies included chronic hepatitis viral infection, primary biliary cholangitis (PBC), cryptogenic cirrhosis, and other causes including alcoholic liver disease, drug-induced liver disease and nonalcoholic fatty liver disease (NAFLD). 

### 3.1. Comparisons of Variables According to the Presence of VH 

Table 1 shows the characteristics between non-VH and VH groups. There were no significant differences in age, sex, etiologies, ALT, AST, TBIL and LS between the two groups (all *p* > 0.05). The PLT and ALB levels were significantly lower in the VH group than in the non-VH group (all *p* > 0.05). Conversely, portal vein diameter, spleen diameter, SWDS and the proportion of ascites were significantly higher in the VH group (all *p* < 0.05).

### 3.2. Factors Associated with VH

Multivariate analysis identified the SWDS, spleen diameter and ascites as the independent factors of VH (Table 1). Based on the multivariate analysis, we derived a formula for diagnosis of VH. 

The regression-based formula (SWDS + spleen diameter + ascites) = −20.749 + 0.804 × SWDS + 0.449 × spleen diameter + 1.803 × ascites (no ascites = 0, ascites = 1).

### 3.3. Diagnostic Performance for VH

**Figure 2:**
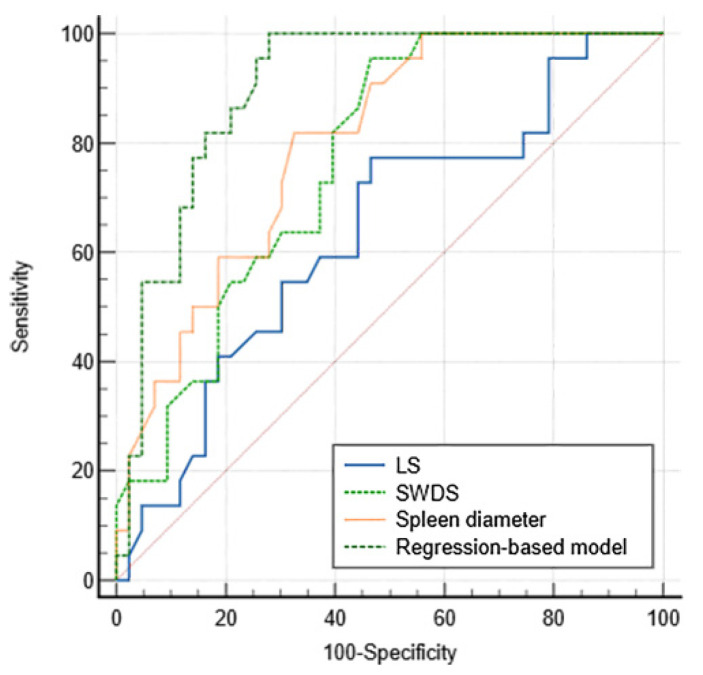
Comparison between AUCs of LS, SWDS, spleen diameter and the regression-based model.

The ROC curves of LS, SWDS, spleen diameter and the regression-based formula (SWDS + spleen diameter+ ascites) are shown in Figure 2, and their diagnostic performances are summarized in Table 2. The AUCs of LS, SWDS, spleen diameter and regression-based formula were 0.637 (95% CI: 0.508–0.753), 0.768 (95% CI: 0.647–0.864), 0.798 (95% CI: 0.680–0.887) and 0.900 (95% CI: 0.800–0.961), respectively. Compared to each independent factor, the regression-based formula demonstrated the highest AUC for diagnosing VH (*p* < 0.001; *p* < 0.05; *p* < 0.05). With a cutoff value of 0.19, the sensitivity, specificity, PPV and NPV of the regression-based formula were 100%, 72.1%, 64.7% and 100%, respectively.

## 4. Discussion

Non-invasive methods for the evaluation of chronic liver disease and its complications include blood-based tests (e.g., serum markers of fibrosis), imaging assessing the anatomy of the liver and other relevant organs, and methods assessing physical properties of the liver (e.g., liver stiffness, LS) [6]. Ultrasound elastography has been developed for assessing LS and shows a good performance in evaluating liver fibrosis. Although LS is mainly determined by liver fibrosis, several confounding factors have been found to influence LS measurement, including liver inflammation, obstructive cholestasis, and hepatic congestion et al. [7,8]. Recent studies have tried to evaluate the utility of LS in predicting the severity of PH and GEVs [6,9]. Some studies have indicated that LS correlates well with HVPG [8]. And the summary AUC for CSPH (defined by an HVPG ≥ 10 mmHg) was 0.90, according to a meta-analysis [10]. However, LS is less accurate for the diagnosis of GEVs than for CSPH [10]. In several studies, the AUCs of LS measured by 2D-SWE for identifying high-risk esophageal varices (HREV) ranged between 0.70–0.88 [11,12,13,14]. Besides, reliable cutoff values of LS are not available yet, no matter for CSPH or for GEVs. Even if the elastography technique is the same, the cutoff values vary across studies [12,13,15]. Above limitations make LS cannot be applied in clinical practice.

The liver is a viscoelastomer with an elastic property, and also a viscous property [16,17]. Both elasticity and viscosity affect the propagation of shear wave. However, most commercial ultrasound elastography techniques neglect the viscosity of the liver and assume it as a pure elastomer [18]. In viscoelastic media, the higher the frequency, the faster the speed. The phenomenon that the shear wave speed is frequency dependent is called dispersion [19]. Thus, we can obtain the dispersion curve from increasing shear wave speeds versus different frequencies. The SWD is a new imaging method developed on the basis of 2-D SWE. By analyzing the dispersion curve, SWD is able to measure the shear wave dispersion slope (SWDS). It is noted that SWDS is not the viscosity coefficient but a parameter indirectly reflecting hepatic viscosity [3]. Viscosity may be an important parameter for characterizing pathological changes of the liver. As a novel technique, there are few studies on SWD, and researchers are trying to explore what SWDS represents pathologically. Several published studies have suggested that SWDS may be related to the degree of hepatic inflammation and fibrosis [18,20]. The persistence of hepatic inflammation leads to fibrosis, which is an important factor associated with the development of PH. Therefore, SWDS may be associated with the presence of VH in cirrhotic patients.

In our study, we found that LS did not differ between patients with and without VH. Although some studies have indicated that LS correlates well with the severity of PH and is a promising tool for the prediction of CSPH [8], it should be underlined that in ranges of HVPG values ≥ 12 mmHg when decompensation events such as VH and ascites develop, the correlation of LS with HVPG becomes less relevant [21]. This suggests that the development of PH does not simply result from the accumulation of collagen fibers. 

In fact, not only fibrosis, but also inflammation contributes to the development and progression of PH. On the one hand, the presence of hepatic inflammation is a prerequisite for the formation of fibrosis, and the structural disturbance of the liver caused by hepatic fibrosis can lead to increased intrahepatic vascular resistance (IHVR). On the other hand, inflammatory mediators and cytokines released after liver injury promote the activation of hepatic stellate cells through a complex process, which subsequently result in an increase of hepatic vascular tone. Dynamic vasoconstriction in sinusoids and portal venules from increased hepatic vascular tone is another important factor causing a rise in IHVR [22]. The increased IHVR to portal blood flow, as a consequence of liver structural disturbance and dynamic vasoconstriction, further causes the development of PH and GEVs [23]. Therefore, hepatic inflammation and fibrosis both play an important role in the occurrence of PH and GEVs. Compared to LS, the SWDS alone showed good diagnostic accuracy for VH (AUC of SWDS vs. LS, 0.786 vs. 0.637, *p* < 0.05). Thus, our study suggests that SWDS could be a useful parameter for evaluating the risk of VH.

The SWDS, spleen diameter and ascites were identified as independent risk factors for VH. In addition, SWDS sensitively identified 95.5% (95% CI: 77.2–99.9%) of patients with VH, with a specificity of 53.5% (95% CI: 37.7–68.8%). When the independent factors were combined, a regression-based formula was developed, which provided a higher specificity (72.1%, 95% CI: 56.3–84.7%). Moreover, our study also indicated that the regression-based formula had a better diagnostic performance with an AUC of 0.900 (95% CI: 0.800–0.961, *p* < 0.001). Splenomegaly is common in patients with cirrhosis, because of blood congestion. Although spleen stiffness (SS) has shown promising results for the detection GEVs and VH in cirrhotic patients [11,24], this study did not explore its value for assessing the risk of VH. Because SS measurement has a lower success rate than that of LS measurement [7,13,25], spleen size is easily measured during abdominal ultrasound examinations. So, we used spleen size to reflect the severity of PH. The presence of ascites is a sign of decompensated cirrhosis, suggesting a higher portal pressure. Therefore, ascites and splenomegaly may also be risk factors for VH. Conventional ultrasound has been a routine examination for assessing ascites and the size of spleen, and when combined with SWDS, it may help improve the diagnostic accuracy of VH. 

The limitations of our study are as follows. First, the sample size was small, so the results are not sufficiently robust. However, as a pilot study aiming to explore whether SWDS is useful, the results of this study have certain interpretability. The reliability of these results needs further research to confirm them. Second, this study enrolled cirrhotic patients with diverse etiologies. Although there was no statistical significance difference in etiologies between non-VH and VH groups, whether the SWDS varies with different etiologies remains unknown. Finally, external validation was not performed owing to the single-center design. Therefore, multi-center, large sample studies are required to validate our results. 

## 5. Conclusions

In conclusion, this study revealed that SWDS could be a sensitive parameter for assessing the risk of VH. When the SWDS, spleen diameter and ascites were combined, a good diagnostic performance was achieved. Thus, SWD appears to be a promising tool for noninvasive evaluation of VH in cirrhotic patients.

## Figures and Tables

**Table 1:** Clinical and Ultrasound Characteristics of the Participants.

Variables	Non VH (*n* = 43)	VH (*n* = 22)	Univariate Analysis		Multivariate Analysis	
			*t/z/χ*²	*p* Value	*OR* (95% CI)	*p* Value
Age (years)	55.65 ± 11.97	58.41 ± 11.73	−0.885	0.380	—	—
Sex			0.210	0.647	—	—
Male	26 (60.5%)	12 (54.5%)				
Female	17 (39.5%)	10 (45.5%)				
BMI (kg/m^2^)	23.69 ± 3.53	22.47 ± 2.36	1.457	0.150	—	—
Etiology			7.434	0.055	—	—
Chronic viral hepatitis	24 (55.8%)	6 (27.3%)				
PBC	11 (25.6%)	5 (22.7%)				
others	4 (9.3%)	5 (22.7%)				
Cryptogenic cirrhosis	4 (9.3%)	6 (27.3%)				
PLT (×10^9^/L)	145.44 ± 59.61	92.59 ± 38.11	3.755	<0.001	—	0.286
ALT (IU/L)	24.80 (17.00–36.20)	20.50 (14.75–43.18)	−0.284	0.776	—	—
AST (IU/L)	28.00 (22.50–43.00)	34.00 (24.00–49.28)	−1.096	0.273	—	—
TBIL (μmol/L)	17.80 (13.00–27.80)	23.95 (16.88–44.45)	−1.879	0.060	—	—
ALB (g/L)	39.96 ± 6.53	36.06 ± 4.90	2.463	0.017	—	0.227
Ascites			9.449	0.002	6.066 (1.287–28.585)	0.023
No	29 (67.4%)	6 (27.3%)				
Yes	14 (32.6%)	16 (72.7%)				
Portal vein diameter (cm)	1.10 (1.00-1.30)	1.30 (1.28-1.40)	−3.578	<0.001	—	0.105
Spleen diameter (cm)	11.48 ± 2.71	14.36 ± 2.00	−4.414	<0.001	1.567 (1.126–2.181)	0.008
LS (kPa)	9.30 (7.40–11.70)	10.60 (9.10–12.68)	−1.796	0.073	—	—
SWDS (m/s/kHz)	15.17 ± 1.88	17.04 ± 1.45	−4.062	<0.001	2.234 (1.267–3.941)	0.005

Note. BMI = body mass index, PBC = primary biliary cholangitis, PLT = platelets, ALT =alanine transaminase, AST = aspartate aminotransferase, TBIL = total bilirubin, ALB = albumin, LS = liver stiffness, SWDS = shear wave dispersion slope, VH = variceal hemorrhage, CI = confidence interval, OR = odds ratio.

**Table 2:** Diagnostic performances of LS, SWDS, spleen diameter and the regression-based model for VH.

Criteria	AUC	*p* Value	Cutoff Value	Sensitivity (%)	Specificity (%)	PPV (%)	NPV (%)
LS	0.637 (0.508–0.753)	0.062	9.5	77.3 (54.6–92.2)	53.5 (37.7–68.8)	45.9 (36.5–55.7)	82.1 (67.0–91.3)
SWDS	0.768 (0.647–0.864)	<0.001	15.2	95.5 (77.2–99.9)	53.5 (37.7–68.8)	51.2 (42.9–59.4)	95.8 (76.9–99.4)
Spleen diameter	0.798 (0.680–0.887)	<0.001	12.6	81.8 (59.7–94.8)	67.4 (51.5–80.9)	56.2 (44.5–67.4)	87.9 (74.5–94.7)
Regression-based model	0.900 (0.800–0.961)	<0.001	0.19	100.0 (84.6–100)	72.1 (56.3–84.7)	64.7 (53.1–74.8)	100.0

Note. Data in parentheses are 95% confidence intervals.

## Data Availability

The data was available only by request.
